# Case presentation-1

**DOI:** 10.1192/j.eurpsy.2026.10268

**Published:** 2026-07-31

**Authors:** A. K. Sikora

**Affiliations:** 1 Ivano-Frankivsk National Medical University, Ivano-Frankivsk, Ukraine; 2 Mazovian Specialist Hospital “Drewnica”, Warsaw, Poland

## Abstract

**Abstract:**

This Clinical Perspectives presentation describes an anonymised case of an adult patient with complex PTSD following prolonged interpersonal trauma, complicated by substance use and problematic benzodiazepine intake. The patient presented with persistent affect dysregulation, hyperarousal, intrusive symptoms, dissociation, sleep disturbance and severe functional impairment, alongside escalating use of alcohol/cannabis and intermittent high-dose benzodiazepine self-medication to manage anxiety and insomnia. The case highlights key clinical dilemmas frequently encountered in trauma-related care: differential diagnosis (complex PTSD vs borderline personality disorder vs major depression), risk assessment including self-harm, and treatment sequencing when trauma symptoms coexist with addiction-related behaviours. Management is discussed using a phased, recovery-oriented approach, integrating stabilisation and psychoeducation, careful psychopharmacological planning (including benzodiazepine harm reduction and tapering strategy), and referral to evidence-based psychotherapy (trauma-focused therapy when clinically safe), combined with addiction-focused interventions and relapse prevention. The presentation emphasises multidisciplinary collaboration and practical decision-making applicable to outpatient psychiatry, particularly when ongoing stressors and limited resources affect continuity of care.

**Disclosure of Interest:**

None Declared

